# Protocol for a pilot cluster randomised controlled trial of PRoGRAM-A (preventing gambling-related harm in adolescents): a secondary school-based social network intervention

**DOI:** 10.1186/s40814-024-01537-w

**Published:** 2024-08-13

**Authors:** Fiona Dobbie, Martine Miller, Heather Wardle, Lucia Dahlby, Christopher Weir, Angela Niven, Andrew Stoddart, David Griffiths, Ashley Lee, Sally Good, Leon Noble, James White

**Affiliations:** 1 Usher Institute, University of Edinburgh, Edinburgh, UK; 2 Edinburgh Clinical Trials Unit, University of Edinburgh, Edinburgh, UK; 3 School of Social and Political Sciences, University of Glasgow, Glasgow, UK; 4 Faculty of Social Sciences, University of Stirling, Stirling, UK; 5 Evidence to Impact, 21B Somerset Square, Nailsea, Bristol, UK; 6 Centre for Trials Research, School of Medicine, Cardiff University, Cardiff, UK

**Keywords:** Gambling-related harms, School intervention, Cluster randomised trial, Process evaluation, Adolescent, Peer-supporters, Social network analysis, Message diffusion

## Abstract

**Background:**

In the UK, recent evidence of young people and gambling indicates a higher prevalence of gambling in comparison to other addictive behaviours. Engaging in gambling-related behaviour at a young age is associated with short and long-term consequences, including financial, emotional, academic, interpersonal, and physical and mental health detriments; otherwise known as gambling-related harms (GRH). Given the unique vulnerability of this younger group, early interventions aimed at delaying or preventing gambling are critical. PRoGRAM-A (Preventing Gambling-Related Harm in Adolescents) is a school-based, social network intervention to protect young people from future GRH, by delaying or preventing gambling experimentation.

**Methods:**

Pilot cluster RCT with an embedded process evaluation and health economic scoping study.

**Participants:**

PRoGRAM-A will be delivered in four schools, with two control schools acting as a comparator. All are secondary schools in Scotland. Baseline surveys were conducted with students in S3 (ages 13–14). Follow-up surveys were conducted with the same cohort, six months post-baseline.

**Intervention:**

PRoGRAM-A trainers will deliver a 2-day, out-of-school training workshop to Peer supporters. Peer supporters will be nominated by peers among their school year group (S3, age 13–14). Workshops will provide peer supporters with information on four gambling-related topics: (1) what is gambling? (2) gambling and gaming, (3) gambling marketing, (4) identifying harm and reducing risk. Peer supporters will disseminate the information (message diffusion) they have learned among their friends and family over a 10-week period. After the 2-day workshop, PRoGRAM-A trainers will conduct × 3 in-school follow-up sessions with peer supporters to offer support, encouragement, and advice to Peer Supporters as well as monitor and explore the extent of their message diffusion.

**Primary outcome:**

The primary outcome of the pilot cluster RCT (cRCT) will be whether progression to a phase III RCT is justified.

**Discussion:**

This will be the first pilot cluster RCT (cRCT) of an intervention to prevent gambling-related harms among young people within the UK. If findings indicate feasibility and acceptability, funding will be sought for a phase III RCT of effectiveness.

**Trial registration:**

Researchregistry8699. Registered 21^st^ February 2023.

**Supplementary Information:**

The online version contains supplementary material available at 10.1186/s40814-024-01537-w.

## Background

Globally gambling is increasingly recognised as a public health concern [1]. The last two decades have witnessed the transformation of the commercial gambling market, with rapid technological advances creating greater opportunity (and ease) to gamble anywhere and anytime. Running parallel to this is the growth of sophisticated and tailored gambling marketing strategies to promote gambling products to consumers. The rapid growth of the gambling industry has been accompanied by a rise in gambling harm, with an estimated six people affected for each person experiencing gambling harm [2].

In response to these associated harms, most countries do not allow children and young people under the age of 18 to engage in commercial forms of gambling [3]. However, children and young people are still exposed to gambling marketing, of which advertising is a key component. A recent UK study reported that 96% of 11–24-year-olds had been exposed to gambling advertising over the last month [4]. A further concern is the intersection of digital gaming and gambling, which has led to young people being increasingly exposed to ‘gambling adjacent’ activities, such as loot boxes, skin betting, social casino games, and sponsorships of e-sports teams by major gambling companies [5]. Despite UK regulatory boards excluding gambling adjacent activities from the definition of gambling [6], young people often identify that they do in fact qualify as gambling [5]. Evidence further suggests that young people who engage in ‘gambling adjacent activities’ are at a greater risk of problematic gambling behaviour [7].

This increased exposure to gambling-related marketing, along with the rise of online forms of gambling has contributed to the normalisation of gambling behaviour within the everyday lives of children and young people. It is, therefore, not surprising that recent systematic reviews of interventions to prevent gambling harm have highlighted children and adolescents as a key priority group and recommend the introduction of more theoretically informed, school-based gambling education programmes, with long-term follow-up [8–10].

In the UK, recent evidence of young people and gambling indicates a higher prevalence of gambling in comparison to other addictive behaviours. For example, a 2022 report conducted by the Gambling Commission found that 50% of 11–16-year-olds have taken part in some form of gambling over the past 12 months, in comparison to drinking alcohol (41%), using e-cigarettes (17%), smoking tobacco cigarettes (7%), or using illicit drugs (5%) [11]. While the incidence of problematic gambling within this age group remains relatively low (0.9%), young people who have taken part in some form of gambling over the past 12 months are more likely to engage in co-occurring risk-taking behaviours, such as alcohol consumption (54%), e-cigarettes and vaping (32%), cigarette smoking (17%), and illicit drug use (16%) [11]. Engaging in gambling-related behaviour at a young age is associated with short and long-term consequences, including financial, emotional, academic, interpersonal, and physical and mental health detriments [2–5]; otherwise known as gambling-related harms (GRH) [12]. Moreover, GRH incurs a considerable economic burden, with a 2023 evidence review by Public Health England reporting the overall economic cost of problem gambling, in the UK, to be between £1.05 and £1.77 billion and the estimated health-related costs to be between £635 and £1355 million [13].

While early intervention programmes exist within public health policy for tobacco, alcohol, and drugs, parallel programs for gambling, separate from industry influence, are lacking. Given the unique vulnerability of this younger group, early interventions aimed at delaying or preventing gambling are critical. This has led to calls for robust, independent early intervention to protect young people from future GRH, by delaying or preventing gambling experimentation [4, 14, 15]. To address this gap, the lead author (FD) obtained funding from the Medical Research Council (MRC) Public Health Intervention Development (ref: MR/S019200/1), to adopt an existing anti-smoking intervention called ASSIST (A Stop Smoking in Schools Trial) that has been successful in protecting young people from smoking harm [16]. This funding supported the development of PRoGRAM-A (Preventing Gambling-Related Harm in Adolescents) and a small feasibility study, which took place in one secondary school between August–November 2021. This protocol paper presents the next phase of PRoGRAM-A’s development—a pilot cluster randomised control trial (RCT). The protocol (current version PRoGRAM-A protocol V2.0 07Aug 2023) is reported using the SPIRIT guidelines [17], with the completed checklist added in Additional file 1: Table S1.

## Methods

### Research aim and research questions

The overall aim is to conduct a pilot cluster randomised control trial (cRCT) of a gambling prevention intervention among young people aged 13–15 to determine the utility of conducting a Phase III RCT assessing effectiveness and feasibility. This aim is unpacked via a series of research questions.

#### Recruitment and randomised trial delivery

1. Can a sufficient number of schools and students be recruited, randomly allocated, and retained?
2. How can the collection of baseline and follow-up data be optimised?
3. What gambling prevention activities occur in control schools and how is the impact perceived?
4. Following the pilot cluster RCT (cRCT), is a phase III cRCT justified in relation to our progression criteria?

#### Acceptability, feasibility, and fidelity of intervention delivery

5) Is it feasible and acceptable to implement the intervention in four schools?
6) What do qualitative and quantitative data suggest in terms of refinements to programme theory, implementation, fidelity, reach, scalability, and acceptability?
7) Are there potential harms and unintended consequences of the intervention? How might these be reduced? How can these be measured?
8) What characteristics are associated with being nominated as a peer supporter?
9) What is the potential and actual extent of message diffusion in peer supporter networks and to whom and why?
10) What contextual factors influence message diffusion (e.g. size of student networks, where, when, and how conversations are initiated, what communication methods are used, what is discussed? level of peer supporter confidence)
11) What are the key issues to consider to support future scalability?
12) What are the direct implementation costs associated with delivering PRoGRAM-A?
13) What economic measures are appropriate and available for use in a future health economic evaluation as part of a definitive cRCT?

### Pilot cRCT

**Fig. 1:**
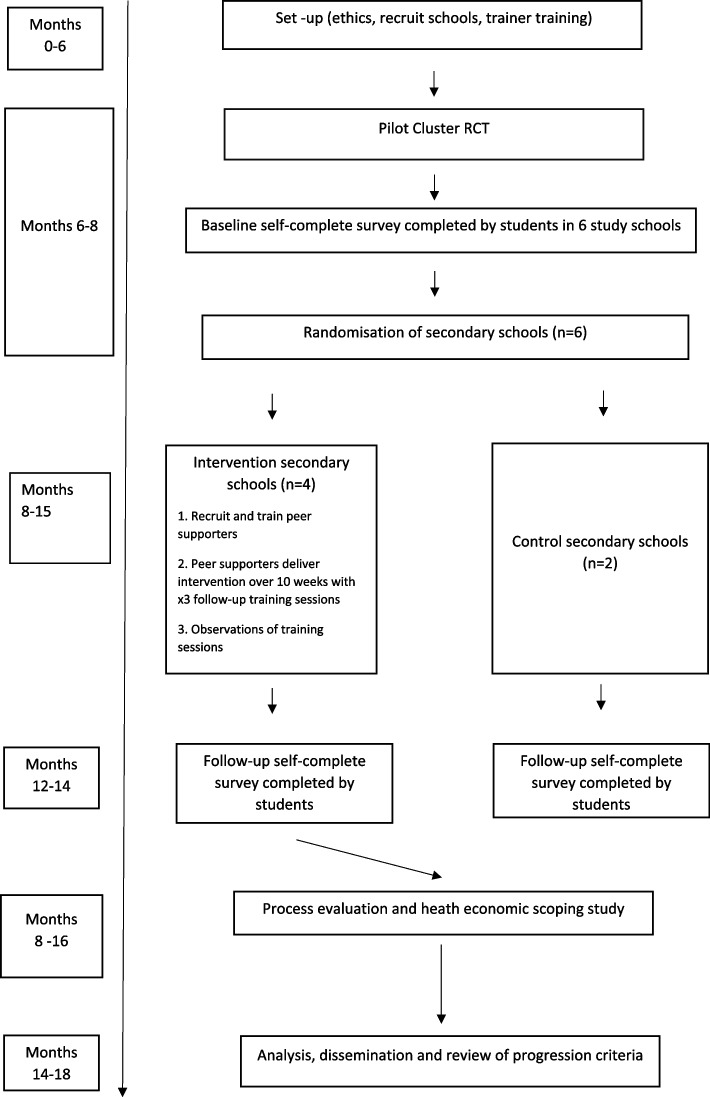
PRoGRAM-A flow diagram

PRoGRAM-A is an 18-month, two-arm, pilot cluster RCT with an embedded process evaluation which includes a social network analysis and health economic feasibility study, conducted in six schools in Scotland. Following the completion of a baseline student survey assessing gambling attitudes, awareness, and behaviour, four schools will be randomised to receive the intervention (PRoGRAM-A) and two will continue with usual practice. The two comparator schools have been included to test the acceptability of randomisation. Key phases of the study are outlined in Fig. 1.

### Setting, inclusion/exclusion criteria

Setting is any state-funded secondary school in Scotland. Inclusion criteria are students in S3, aged 13–15, (equivalent to years 9 and 10 in England, Wales, and Northern Ireland) who give their assent to participate in the study. The exclusion criteria are (1) schools for young people with special needs, (2) residential schools, (3) students who do not provide assent, (4) student’s parent/carer who opt their child out of the study or do not give consent for peer supporter training, and (5) schools who do not consent to participate.

### Population

Informed by findings from our development study, students in S3 were chosen because it is an age when experimentation in risk-taking behaviour can start and social norms are established [18]. Consultation with schools and teaching staff also suggested that S3 was an appropriate year due to the young people being more mature and able to understand and engage with different components of PRoGRAM-A (e.g. marketing strategies used by the gambling industry). Finally, from a practical perspective, S3 is the year before students sit their National 4 exams, meaning it would be less disruptive to the school and students.

### Sample

As this is a pilot cRCT (to determine progression to a phase III RCT or not) a power calculation was not required. Six schools across Scotland will take part in the study, four of which will be assigned to the intervention and two of which will be assigned to the control. Four intervention schools balance the need to ensure there is a diversity of schools to inform the delivery of a future phase III RCT but ensure the pilot is small enough to be cost-effective. Depending on the size of the school, the year group size for S3 students could vary between 100 and 150 students. Using a conservative assumption of 85% [16] for attendance on the day of the baseline survey and consent to participate, this gives a projected sample size for the baseline survey of between 510 and 765 students. This range of sample size will enable the proportion of enrolled students completing the baseline questionnaire to be estimated with a 95% confidence interval width ± 2.3% to ± 2.9%, informing planning of the definitive trial.

### Recruitment

Schools will be invited to take part in an online information webinar where they can find out more about PRoGRAM-A and what taking part in the pilot RCT would involve. This will help schools make an informed decision about whether they would like to volunteer to take part or not. The school’s webinar invitation will be circulated via existing networks from the research team. This includes dissemination via two voluntary sector organisation (Fast Forward and Larkhall Universal Connections) that have delivered existing gambling prevention work in several schools throughout Scotland. At this pilot stage, there is no restriction on school location, with any state-funded school in Scotland eligible to express interest in taking part.

After the webinar, schools who are interested in finding out more information will email a member of the research team. Depending on the number of schools that express an interest, a purposive sampling frame may be applied to ensure diversity in terms of school size and location. Schools that opt in to PRoGRAM-A will then receive a site visit from a member of the research team where they will be given a more detailed briefing about what is involved and have the opportunity to ask questions. After this site visit, and if they still want to proceed, the school will be registered into the pilot RCT.

Students will be given an information letter about the survey at least 1 week prior to data collection and an oral description of the study from school teachers. Students will have the opportunity to ask questions before deciding whether they want to take part or not. On the day of data collection, students will be invited to give signed assent to participate in data collection. In advance of data collection parents/guardians will be sent an information letter telling them about the research study and giving them an opportunity to opt their child out if they do not want them to participate.

### Randomisation

Schools will be randomised using a remote system set up by Edinburgh Clinical Trials Unit in order to conceal the allocation sequence. The allocation sequence will be stored on a secure server and concealed from all personnel involved in the trial. It will be created, using computer-generated pseudo-random numbers, by a clinical trials unit member of staff with no link to, or contact with, any of the participating schools. The randomisation will be stratified by school size (less than 200/greater than or equal to 200 students on the 3rd year school roll). The PRoGRAM-A intervention to comparator group randomisation ratio within each stratum is 2:1.

Once all schools have been identified, confirmed they wish to take part, and baseline forms have been completed, a designated member of the trial team will contact the Edinburgh Clinical Trials Unit (ECTU) data management team requesting the randomised assignment of all schools. These assignments will then be communicated to the schools by the trial team member. Prior to baseline form completion all trial team members, schools, and students will be blinded; after this point, all will be unblinded.

### Planned intervention

**Fig. 2:**
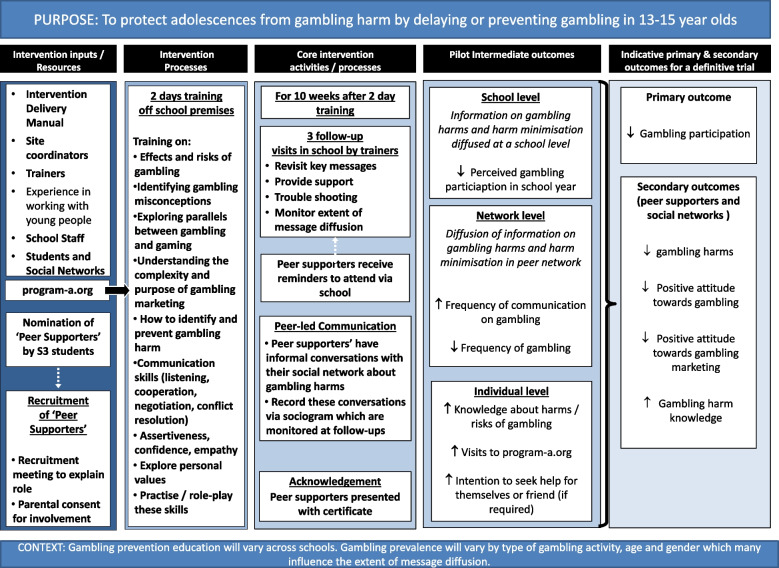
PRoGRAM-A logic model

PRoGRAM-A defines gambling as the participation in betting or wagering of things of value (including but not limited to, flat currency, digital currency, and objects of value), which includes all commercial forms of gambling (ranging from lotteries, scratch-cards to online casinos and betting) and gambling-adjacent activities like loot boxes, skin betting, and social casino gambling. It is an adaptation of an effective school-based, peer-led smoking prevention programme, A Stop Smoking in Schools Trial (ASSIST) [14, 15]. As illustrated in our logic model (Fig. 2) the aim of PRoGRAM-A is to identify ‘opinion leaders’ in S3 who are trained to become ‘peer supporters’. Peer supporters are then trained to have informal conversations with anyone in their social networks about the risks of gambling, the influence of gambling marketing, and the links between gaming and gambling [17].

PRoGRAM-A is theoretically grounded in diffusion and network intervention theory. Diffusion theory (also referred to as diffusion of innovation theory), explains how new ideas and social norms are introduced and spread throughout communities [18]. The application of diffusion theory in intervention design relies on identifying influential people who have expertise and credibility among their peers to promote/create new social norms [17]. Social networks are, therefore, a crucial component to support the delivery of diffusion theory. Table 1 summarises the PRoGRAM-A intervention using the ‘Template for Intervention Design and Replication’ (TIDiER) checklist [19].

**Table 1:** Description of PRoGRAM-A Intervention using TIDieR checklist

TIDier item	Information on PRoGRAM-A intervention
Brief name	PRoGRAM-A intervention
Why	In the UK, recent evidence has shown that there is a higher prevalence of gambling in young people compared to other addictive behaviours. Early intervention programmes exist for tobacco, alcohol, and drugs, however, similar programmes for gambling are lacking. PRoGRAM-A is theoretically grounded in diffusion and network intervention theory. The application of diffusion theory in the intervention design relies on identifying influential S3 students who have credibility among their peers, who once trained, will have the ability to promote gambling prevention messages and raise awareness of gambling harm in their social network/community
What materials	Peer Supporters will receive 2 days of training, delivered out of school in a community venue. The training is developed from the intervention delivery manual. The catering and venue costs (if any) will be covered by the study. Pin badges and banner pens will be provided for students undergoing the peer supporter training
What Procedures	PRoGRAM-A is a peer-led gambling prevention programme for S3 students. Students will complete a peer nomination questionnaire, nominating those within their year group they respect or look up to. The students with the highest number of nominations will be invited to be peer supporters. At the time of peer nomination, students are not aware that they are identifying influential others in a gambling-related context to prevent biased nomination. Peer supporters undergo 2 days of training covering: (1) what is gambling; (2) gambling and gaming; (3) gambling advertising and marketing; (4) gambling-related harm and keeping safe. They learn how to have informal conversations with anyone in their social network about these topics. These conversations aim to spread new social norms throughout their network and community. After the initial training, the trainers will meet with the peer supporters at three follow-up sessions over the next 8 to 10 weeks to check their progress and to maintain engagement
Who provides	Implementation of the intervention including the 2-day training and the follow-up sessions will be provided by trainers from ‘Evidence to Impact’—a social enterprise that specialises in translating public health into practice. They will be supported by the research team and school staff to arrange suitable times/accommodations for the training and to gather pupils together for the follow-up sessions as well as the interviews and focus groups as part of the process evaluation (all during school hours). Two members of the teaching staff will accompany the peer supporters to the training venue—they will not be involved in the training. They will act as chaperones and will only be called upon if pupil behaviour becomes challenging
How	Each school will identify a main point of contact within the school for the study. The research team will liaise with the school and the trainers to identify and book a suitable training venue as close to the school as possible (ideally walking distance). Transport, if required, will be organised by the research team and trainers with input from the schools. The school will identify staff to accompany the students to the venue and to act as chaperones for the 2 days. The research team will also liaise with the school (and trainers) to book class space and time for the peer supporters to attend the three follow-up sessions and any interviews/focus groups that students are invited to take part in
Where	The initial 2-day training of Peer Supporters will be carried out away from school premises usually in a community venue. Follow-up sessions with Peer Supporters will take place in school during class time. The Process Evaluation interviews/focus groups involving school students will take place in school and during school hours. Interviews with teachers, trainers, and peer supporter’s adult friends/family members can be conducted face-to-face or over the phone/MS Teams at a time/place convenient to the interviewee
When and How Much	Training by ‘Evidence to Impact’ will be carried out over two school days with Peer Supporters. The three follow-up sessions will take place in school during one school period/module 40–50 min each. These will be held at regular intervals over the 8 to 10 weeks after the peer supporter training. Interviews and focus groups will be held after the intervention has been completed and are likely to last between 50 and 60 min depending on how much people have to say
Tailoring	Discussions will take place at each school regarding students with additional support needs and how the intervention can be adapted to make sure all students can be included
How well (planned fidelity assessment)	A process evaluation will provide an assessment for fidelity via: • Recordings of semi-structured interviews and focus groups with peer supporters and other S3 students • Semi-structured interviews with peer supporter’s friends and family members • Semi-structured interviews with teaching staff, intervention trainers, and stakeholders • Observations of two entire delivery cycles of PRoGRAM-A in two separate schools, using semi-structured observation log books

### Comparator

To better understand the influence of peer supporters in the dissemination of gambling prevention and gambling harm messages among youth, we will measure outcomes across six state-funded secondary schools in Scotland randomised to the PRoGRAM-A intervention or to continue with usual practice (recorded before baseline survey completion). The control group school may or may not include the provision of GRH education in school. Although there is no requirement for the provision of gambling prevention education within secondary schools, some have ‘ad hoc’ lessons. If these are present, the information provided will be described in the process evaluation.

### Outcome measures

The primary outcome for this pilot trial is whether progression to a full-scale phase III cRCT of the intervention is warranted. This will be assessed against a set of pre-specified progression criteria (Table 2). Our proposed primary outcome measure for a future phase III cRCT is self-reported gambling participation (measured by asking about types of gambling participation ‘in the last 4 weeks’ and ‘in the last 12 months).

**Table 2:** PRoGRAM-A progression criteria

Progression criterion	Red	Amber	Green
1. Successful recruitment of six schools	< 6		6
2. Five schools remain in the pilot study	< 4	4	≥ 5
3. The intervention being delivered with 80% fidelity to the manual	≤ 69%	70–79%	≥ 80%
4. The process evaluation indicates the intervention is acceptable to students and staff	Low	Medium	High
5. 70% of students complete the student questionnaire at baseline and follow-up	≤ 59%	60–69%	≥ 70%

### Progression criteria

Progression criteria are presented in Table 2 using the red, amber, and green traffic light system and will be monitored by an independent Trial Steering Committee (TSC). If criteria in the red category are not met the study will not progress to a definitive trial. If criteria meet the amber category further discussion will be required with the TSC regarding progression and actions required to support this. Criteria that meet the green category indicate favourable progression to a Phase III trial.

#### Assessment and follow-up

Baseline data collection will be conducted before the randomised allocation to PRoGRAM-A or control is revealed to schools. A baseline assessment will be completed in the first term of school with a follow-up survey six months later. Given the logistical challenges of conducting fieldwork in schools, data collection will be paper-based, ideally conducted under exam conditions via a special assembly. Students will be reassured that completing the survey will be completely confidential and anonymous with their name not attached to the survey. Members of the research team and class teachers will be present to help administer the survey, answer any questions students may have, and also support students with additional needs.

Peer supporters will receive three follow-up visits from the trainers. Conducted in school these visits will collect data on the extent of message diffusion among their social network, reinforce key messages, and address any questions or challenges peer supporters may have. Trainers will record any unintended consequence either through observation or direct feedback from school staff and students.

### Statistical analysis

Results from the study will be reported using the CONSORT guidelines for pilot and cluster RCTs. The proportion (and exact binomial 95% confidence interval, CI) of students assenting to participate will be estimated, overall and stratified by school. The proportion of students (and exact binomial 95% CI) completing baseline and follow-up questionnaires will be reported overall and by the school. We will summarise the demographic characteristics of students by randomised group and by the school using descriptive statistics.

For each of the quantitative outcome measures on gambling participation, harms, knowledge, and attitudes, the proportion of missing data will be reported overall, by intervention group and by school. Data will be summarised descriptively recording the potential for floor and ceiling effects. We will then pilot the analyses of outcomes that would be performed in a full-scale trial. Outcomes at follow-up will be analysed by multi-level regression modelling, adjusting for baseline values. Estimates for differences between intervention and control (odds ratios and least squares mean differences) will be adjusted for clustering (students nested within schools) and presented alongside 95% CIs. We will also make preliminary estimates of the clustering of outcomes within schools by estimating intra-cluster correlation coefficients (and 95% CIs). As this is a pilot trial, not powered for effectiveness, no hypothesis testing will be performed and no *p*-values presented.

A detailed statistical analysis plan will be prepared, blinded to randomised intervention allocations and accumulating trial data, and finalised prior to database lock.

### Process evaluation

Following the MRC guidelines for process evaluations of complex interventions [20] with a focus on implementation, a mixed methods process evaluation will be included. This will examine: intervention feasibility, fidelity, reach, and acceptability; record the provision of education (if any exists) on GRH in all six schools to assess potential contamination and; explore context and potential mechanisms of action, including unintended effects. It will also inform the health economic scoping study and the social network work analysis.

### Health economic evaluation

A health economic scoping study will be integrated with the process evaluation. Stakeholder consultation exercises will be built into the qualitative interviewing to identify economic outcomes of interest to different stakeholder groups with a view to developing recommendations for cost-consequence and/or social return on investment analyses for a phase III future trial. Additionally, an activity-based costing exercise will identify direct intervention delivery costs relating to direct staff time, equipment, and materials for training sessions plus overheads and any additional items identified through consultation with associated staff. The results of the stakeholder consultation and the activity-based costing exercise will be combined into a short report describing the proposed evaluation methods.

### Qualitative analysis

All Interviews and focus groups collected during the process evaluation will be digitally recorded and transcribed verbatim. We will use an inductive, thematic content approach to analyse the data [21] (including observational data), facilitated by NVivo 14. First, we will read the transcripts to identify the key themes and sentiments that emerge from the data. Then a draft analytical framework will be created, piloted, refined, and finalised by the research team. Each transcript will then be coded and summarised into key themes using framework matrices or charts. This approach reduces large volumes of data and facilitates systematisation between and within-case analysis. The use of NVivo 12 ensures that analysis is fully documented and conclusions can be clearly linked back to the original source data.

### Social network analysis

Social network data collected at the peer supporters’ training and follow-up will be used to create an anonymised ego network for each peer supporter which will collect information on people they could/have spoken to (e.g. age, gender, school year, ethnicity), whether a conversation had taken place and an assessment of perceived impact. The selection will be explored based on personal (socio-demographic) and network (centrality/clusters within ego-network) characteristics to ascertain the scope of the intervention in terms of reach and equality of access. Linking the follow-up questionnaire to the peer nomination questionnaire will enable diffusion across the whole year group to be assessed, including differences between socio-demographic groups. The effectiveness of peer supporter selection will be assessed by mapping respondents’ ego networks (such as density and centralisation), and positioning individuals talked to against perceived outcomes to assess who has been reached. This is in an effort to provide insight for future studies on how to exercise best practice in the selection of peer supporters to identify inequalities in the reach of the intervention. Such analyses will also help determine overlap in social connections, providing estimations of the likely crossover of receiving the intervention amongst the wider community.

### Ethics, research governance, and trial registration

Ethical approval will be obtained from The University of Edinburgh Medical School Research Ethics Committee, which is compliant with the ethics framework set out by the UKRI, Economic and Social Research Council. ACCORD (Academic and Clinical Central Office for Research and Development) is a partnership between the University of Edinburgh and NHS Lothian Health Board and as our sponsor will provide advice and support throughout the study. Local Authority Education approval will be required to engage with secondary schools. All members of the research team conducting research in schools will require a Protection of Vulnerable Groups Disclosure Scotland check. The pilot RCT has been registered with the Research Registry on www.researchregistry.com (reference:8699). The minimum level of personal data will be collected. All data will be stored on the University of Edinburgh’s networked storage space, DataStore. DataStore is accessed via password-protected desktops or encrypted laptops. Files holding sensitive information such as raw data or participant information will be held in an encrypted project folder on DataStore. Once data collection is complete and transcripts checked, interviews and focus group discussions will be deleted. All participant paperwork will be stored in a locked drawer in the research team’s locked office (pin entry system). All trial-related documents will be archived for 5 years in accordance with the Sponsor’s archiving policy and then destroyed.

### Study management

The Chief Investigator (CI), Fiona Dobbie, will have overall responsibility for the conduct of the study. The day-to-day management of the RCT will be coordinated by Angela Niven the project manager. The Trial Management Group (TMG) consisting of co-investigators, collaborators, and CTU staff will meet monthly. The Trial Steering Committee (TSC) will meet three times over the duration of the study and will advise on its conduct and progress. Progression criteria set by the funder (NIHR) will be monitored by the TMG and the TSC.

### Stakeholder engagement

A young person’s stakeholder engagement group will be set up prior to the delivery of the PRoGRAM-A intervention. The group will consist of 6–10 young people aged 12–15 years old. The purpose of this group will be to review the four topics that will be delivered by the PRoGRAM-A trainers: (1) what is gambling; (2) gambling and gaming; (3) gambling advertising and marketing; (4) gambling-related harm and keeping safe. In addition to the four topics, the group will also provide feedback on the PRoGRAM-A student survey and study website.

These feedback sessions will be an opportunity for young people to feed into the development of the PRoGRAM-A training manual by ensuring that the topic content and related activities are engaging, relevant, and comprehensible. The group will be facilitated by trained youth workers with experience in designing and delivering interventions to raise awareness of gambling harms among young people. Members of the research team will also attend the PPI sessions to make observations and note relevant feedback.

## Discussion

A large proportion of the global research on gambling and responsible gambling programmes is funded by the gambling industry [22, 23]. The existing evidence base is further criticised for its focus on pathological, disordered gambling which neglects the broader and more complex impacts on society. If successful, PRoGRAM-A has the potential to create new knowledge by being one of the first evidence-based, independent interventions to prevent GRH in young people. Findings will be disseminated via a peer-reviewed publication, conference presentation, and summary reports, to a range of audiences, using social media and university websites.

As informed by a previous development study, this pilot cluster RCT of PRoGRAM-A will generate knowledge about whether conducting a phase III cRCT is warranted. We will pilot test delivery of baseline and follow-up paper surveys in secondary schools and conduct a process evaluation to measure acceptability and feasibility as well as intended and unintended outcomes. The health economic and scoping study will consult with key stakeholders to assess what data is required to inform decisions about future rollout and scalability. The social network study will provide important detail around the potential and actual extent of message diffusion as well as the context around who conversations were with, where they took place, and mode of communication. Using pre-defined progression criteria, the Trial Steering Committee will determine whether progression to a phase III RCT is warranted or not.

## Strengths and limitations

A key strength of this pilot RCT is its randomised design, stratification by school size, and its delivery in secondary schools which is a replicable and scalable setting for population-wide intervention delivery. Further, the preceding development study (ASSIST) created links with local and national governments as well as the education, public, and voluntary sectors which can help to facilitate the future rollout of PRoGRAM- across schools in the UK, should findings from the proposed pilot study support a definitive trial. Finally, PRoGRAM-A has the potential to be cost-effective (a key factor in scalability and translation) due to reliance on social capital and spreading information through social networks thus maximising reach and potential effectiveness [17].

However, there are three key limitations. The small number of pilot schools makes it difficult to pilot different combinations of school variables such as schools in urban/rural areas and schools in deprived/affluent areas. Next, whilst a lot of the delivery costs will be ‘in kind’ contributions rather than an actual financial payment, funding will be required to support the delivery of PRoGRAM-A (e.g. payment of youth worker time, venue hire, and catering costs for peer supporter training) and quality assessment to maintain intervention delivery integrity. Finally, despite a key strength of PRoGRAM-A being its peer approach (with students nominated by their peers, as opposed to teachers), once a peer-supporter is nominated they may choose to either accept or decline the role. The option for students to revoke their participation could result in the effects of attrition bias as more confident, outgoing students may self-select to remain, while more introverted students may choose to decline. However, this has never been reported in ASSIST or any other adaptations to the programme. In order to combat the potential effects of sampling and attrition bias, the study population will be continually monitored and discussed with the research team in order to control for any unintended effects.

### Supplementary Information

Additional file 1: Table S1. SPIRIT 2013 Checklist: recommended items to address in a clinical trial protocol and related documents

## Data Availability

Not applicable.

## Abbreviations

ACCORD: Academic and Clinical Central Office for Research and Development
ASSIST: A Stop Smoking in Schools Trial
CRCT: Cluster Randomised Control Trial
ECTU: Edinburgh Clinical Trials Unit
GRH: Gambling-related harm
MRC: Medical Research Council
PRoGRAM-A: Preventing Gambling-Related Harm in Adolescents
RCT: Randomised control trial
TIDiER: Template for Intervention Design and Replication
TMG: Trial Management Group
TSC: Trial Steering Committee
